# Improving patient experiences of mental health inpatient care: a randomised controlled trial

**DOI:** 10.1017/S003329171700188X

**Published:** 2017-07-20

**Authors:** T. Wykes, E. Csipke, P. Williams, L. Koeser, S. Nash, D. Rose, T. Craig, P. McCrone

**Affiliations:** 1 Institute of Psychiatry, Psychology and Neuroscience, King's College London, London, UK; 2 South London and Maudsley NHS Trust, London, UK; 3 Division of Psychiatry, University College London, London, UK; 4 Institute of Psychiatry, Psychology and Neuroscience, Kings College London, London, UK; 5 London School of Hygiene and Tropical Medicine, Keppel Street, London, UK; 6 Service User Research Enterprise, Institute of Psychiatry, Psychology and Neuroscience, King's College London, London, UK; 7 Health Services and Population Research Department, Institute of Psychiatry, Psychology and Neuroscience, King's College London, London, UK; 8 Health Economics, Health Services and Population Research, Institute of Psychiatry, Psychology and Neuroscience, King's College London, London, UK

**Keywords:** Inpatient wards, mental health, patient perceptions, randomised trial

## Abstract

**Background:**

Poorer patient views of mental health inpatient treatment predict both further admissions and, for those admitted involuntarily, longer admissions. As advocated in the UK Francis report, we investigated the hypothesis that improving staff training improves patients’ views of ward care.

**Method:**

Cluster randomised trial with stepped wedge design in 16 acute mental health wards randomised (using the *ralloc* procedure in Stata) by an independent statistician in three waves to staff training. A psychologist trained ward staff on evidence-based group interventions and then supported their introduction to each ward. The main outcome was blind self-report of perceptions of care (VOICE) before or up to 2 years after staff training between November 2008 and January 2013.

**Results:**

In total, 1108 inpatients took part (616 admitted involuntarily under the English Mental Health Act). On average 51.6 staff training sessions were provided per ward. Involuntary patient's perceptions of, and satisfaction with, mental health wards improved after staff training (N582, standardised effect −0·35, 95% CI −0·57 to −0·12, *p* = 0·002; interaction *p* value 0·006) but no benefit to those admitted voluntarily (N469, −0.01, 95% CI −0.23 to 0.22, *p* = 0.955) and no strong evidence of an overall effect (N1058, standardised effect −0.18 s.d., 95% CI −0.38 to 0.01, *p* = 0.062). The training costs around £10 per patient per week. Resource allocation changed towards patient perceived meaningful contacts by an average of £12 (95% CI −£76 to £98, *p* = 0.774).

**Conclusion:**

Staff training improved the perceptions of the therapeutic environment in those least likely to want an inpatient admission, those formally detained. This change might enhance future engagement with all mental health services and prevent the more costly admissions.

## Introduction

People who perceive inpatient mental health care negatively are more likely to require a further admission under a legal sanction (Csipke *et al.* 2014; van der Post *et al.* 2014) such as the English Mental Health Act (MHA). Those subsequently readmitted also have poorer therapeutic relationships and service engagement and their admissions tend to be longer by about 70 days and are therefore more costly (Williams *et al.* 2014). Given that engagement and therapeutic relationships are important for all patient outcomes, improving the experience of inpatient care is a key target for all, but particularly for those who do not accept inpatient services and are admitted involuntarily under an MHA legal sanction. Long before the Francis Report (Francis, 2013) highlighted grave shortcomings in inpatient care, concerns had been raised about the poor quality of services in mental health. The most recent report by the UK Care Quality Commission (2015) painted a bleak picture of mental health inpatient care, particularly the increasing numbers of people detained and compulsorily treated. This cycle of poor perceptions of inpatient care and increasing numbers of people compulsorily treated might be broken if we can find cost-effective ways to improve the inpatient therapeutic environment, which then has an effect on patient perceptions.

Many patients and frontline staff themselves complain about the quality of psychiatric inpatient care, often citing the concern that there is very little to do which results in intense boredom (Mind, 2017, Ward Watch: Mind's campaign to improve hospital conditions for mental health patients, Star Wards, 2014). This is not a purely UK phenomenon as professional organisations and patient advocacy groups internationally (e.g. Mental Health Council of Australia, US National Alliance on Mental Illness) recommend that patients should have access to 4 h/week of therapeutic activities in inpatient settings in addition to one-to-one staff contact (Cresswell *et al.* 2014). Our earlier cross-sectional study (Csipke *et al.* 2014) of patient perceptions of ward care found that activity and one-to-one sessions with staff were associated with better perceptions of care. Like others we also found, unsurprisingly, that satisfaction with care was poorer in those who were compelled legally to accept inpatient care (Katsakou *et al.* 2010; Smith *et al.* 2014; van der Post *et al.* 2014). Although there have been some successful attempts at introducing activities (Hansen & Slevin, 1996; Dodds & Bowles, 2001), nurses still report the primary reason for not spending time on therapeutic activities or direct patient contact is the need to resolve crises, increasing administration and their perception of a lack of skills necessary to implement evidence-based activities (Ward & Cowman, 2007; Seed *et al.* 2010).

Our intervention consists of providing a supported staff training programme for evidence-based therapeutic activities. We thought that this may redress the skill shortage, build self-confidence in staff and encourage more staff contacts and activities. We hypothesised that all these effects should benefit patients’ perceptions of the therapeutic environment and that was also the view of our service user collaborators. The pathway from intervention to impact is complex by including improvements in staff morale, changes in activities, provision of opportunities for patients to attend as well as effects on patients themselves. None of these effects are mutually exclusive. We therefore tested the simple effect of whether staff training changes patient perceptions of the therapeutic environment. Because patients who had been admitted under a legal section have much poorer perceptions of care and poorer outcomes, we specifically hypothesised that the staff-training intervention would have effects on the perceptions of this group.

The study aims were:

• To investigate patient perceptions of ward care following staff training and support for ward-based therapeutic activity and specifically investigate the effects on involuntarily admitted patients.
• To examine the impact of the programme on patients’ perceptions of the amount of care received, particularly those admitted involuntarily.
• To examine the costs of this care.

## Methods

### Study design and participants

**Fig. 1.**
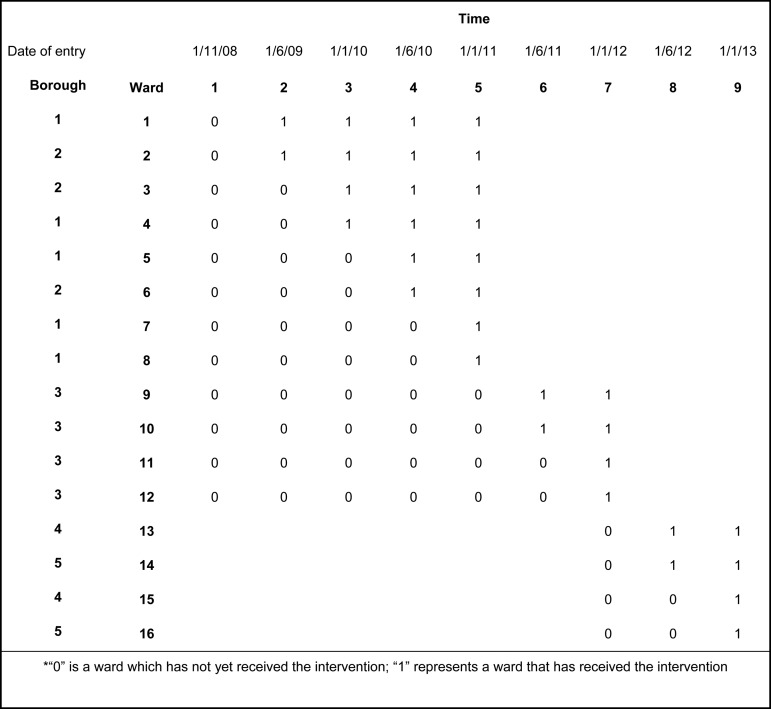
Intervention schedule*

The study was a stepped wedge design (Hayes & Moulton, 2009), which is a type of cluster randomised trial where the timing of the intervention is randomised so wards randomised to receive staff training remained in the intervention arm subsequently. All participants entered the dataset once only even if they were readmitted during the study and so provided only one set of data in either the pre- or post-intervention period. They were unaware of the condition to which they were allocated, so all main outcomes were blind rated. Wards were sampled 3, 5 or 7 times (see Fig. 1). Patients were eligible if they could communicate in English, had been on the ward for a minimum of 7 days and could provide informed consent. The only exclusion criterion was previous participation in the trial. We endeavoured to recruit 50% of all eligible patients at the time of data collection. This study was carried out in distinct geographic areas (‘Boroughs’) and details are given in panel 1. Ethical approval was granted by Bexley and Greenwich Research Ethics Committee (Ref: 07/H0809/49).

**Panel 1.** The setting

• ***Borough 1*** serves an inner city population that has a high deprivation index. Five 18-bedded wards participated in this study, three for men and two for women.
• ***Borough 2*** serves a more suburban affluent area. Three wards participated in our study, one for men and two for women. Two wards had 22 beds and one, a women's ward, had eight beds and did not admit patients under any legal sanction.
• ***Borough 3*** has a high deprivation score and four 18-bedded wards provide acute inpatient care. There were two single gender and two mixed gender wards (a triage ward and an early intervention unit).
• ***Borough 4*** was mixed socioeconomically with two 18-bedded mixed gender wards serving an area with a high deprivation score.
• ***Borough 5*** serves a suburban and affluent area and had two mixed gender, 18-bedded wards.

## Randomisation and masking

Wards were randomised two at a time to the intervention, which for pragmatic reasons was performed in three waves (eight wards in first wave, four in the second and four in the final wave). Randomisation was carried out separately within boroughs by an independent statistician using the *ralloc* procedure in Stata. After baseline, the first two randomised to intervention wards received staff training, with a further two wards randomised every 6 months until all wards had received the training (see Fig. 1).

## Outcomes

### Participant level data

#### Main outcome

*Views on inpatient care* (VOICE; Evans *et al.* 2012) is a 19-item multi-faceted self-report measure developed with service user involvement via participatory methods with good reliability and validity. VOICE measures trust and respect received from ward staff as well as therapeutic contact and care. The main outcome is the total score (range 19–114) where higher is a worse perception of care.

#### Secondary outcome

*Service satisfaction scale*: residential services evaluation (SSS-Res; Greenfield & Attkisson, 1989, 2004). SSS-Res is a 33-item measure that concentrates more on the physical environment than VOICE and has been used in other studies of inpatient care (e.g. Osborn *et al.* 2010). The key outcome was the total score (range 33–165) and again a high score is a worse perception.

#### Other clinical measures

(i) *Positive and negative syndrome scale* (PANSS; Kay *et al.* 1987): All trained raters achieved parity with the key expert rater on a ‘gold standard’ video (item scores within 2 points on 80% of the items). The key outcome was the total score (range 30–180).
(ii) *Nurses Observational Scale for Inpatient Evaluation* (NOSIE; Honigfel *et al.* 1966) is a 12-item nurse rated scale focusing mainly on socially unacceptable behaviour in an individual patient over the past week. The key outcome was the total score (range 0–44) and a higher score is worse behaviour.

#### Resource measures

*Client services receipt inventory-inpatient* (CITRINE; Sabes-Figuera *et al.* 2012) assesses, by patient report, how much meaningful contact was made with ward staff and their engagement in activities over the past week, which enables the calculation of the cost of such engagement using unit cost data (Curtis, 2012).

#### Descriptive data

*Participant's background information* included age, gender, ethnicity, primary diagnosis, first language, length of stay (up to entry into the study) and whether they were detained under a legal sanction. *Ward level data:* In addition to the average acute psychiatric problems experienced by patients in a ward (indexed by the average NOSIE and PANSS scores), we also captured the number of ward activities and how many individuals attended these from evidence logged by the ward and compared average frequencies before and after the intervention.

### Staff-training intervention

Following consideration of NICE guidelines and with a consultation team consisting of trust clinical leads, ward managers and nursing staff directly involved with each ward, eight activities were chosen, based on evidence of feasibility and acceptability to ward staff, and where training input was relatively modest and usually available in the NHS. Not all interventions could be provided on a single ward at the same time, so four were chosen by the consultation team to be core training. The staff training sessions involve different health care professionals and were provided when those staff were available. Training offered to all wards: (i) Social Cognition and Interaction Training (Penn *et al.* 2007), (ii) CBT-based communications training for nurses (co-facilitated by a service user educator) and (iii) computerised Cognitive Remediation Therapy (to involve Occupational Therapists) (Reeder *et al.* 2016), (iv) Pharmacists were recruited to run medication education groups (Kavanagh *et al.* 2003). Ward staff could choose more sessions according to individual ward needs from: Hearing Voices Group (Ruddle *et al.* 2011), Emotional Coping Skills Group (Linehan, 1997), Problem Solving Skills (Grey, 2015), Relaxation/Sleep Hygiene and Coping with Stigma Group (Knight *et al.* 2006). The staff training intervention was provided after randomisation and was both off site and in vivo. Following the training workshops the trainer, a clinical psychologist, provided supervision during the intervention period, which consisted of weekly visits initially but then reduced and depended on the activity and the staff skills. Most supervision had been completed 6 months after the initial training workshop. Details of the staff training can be found in online web Table 1 and training materials can be found on the study website (http://www.perceive.iop.kcl.ac.uk/).

### Procedures

Researchers approached all eligible patients and participants gave written informed consent. Recruitment lasted for a period of 4 weeks at each assessment point.

### Statistical power and analysis

The assumed total number of measurements was 16 wards with 15 patients per ward sampled over three time points as a minimum after baseline, i.e. a total of 720. As an approximation, we treated the design as a standard cluster randomised trial with clusters of size 30 (two wards of size 15 were randomised in pairs) with an estimated intraclass correlation of 0.05 following a conservative approach using data from Adams *et al.* (2004).This sample size in a standard cluster randomised design would have given approximately 90% power to detect a standardised effect size of 0.5 (moderate), using double-sided significance tests with *α* = 0.05. (There was no additional clustering at the patient level as the sample differed at each time point). Because of the stepped wedge design, the actual number of wards and participants in the intervention and control groups varied according to time point, so the above calculations are approximate, but were designed to be conservative.

#### Effects directly on patients

For all measures obtained by self-report it was a requirement that at least 80% of the questions were completed to be included.

(*i*) *Individual patient participants*: We ran two analyses for all primary and secondary outcomes using linear regression adjusting for time and ward as fixed effects and then we additionally adjusted for any identified confounders (defined as a variable associated with both treatment and the outcome with a *p* value < 0.1. Potential confounders considered were gender, age, ethnicity, diagnosis, number of previous admissions, inpatient days on current admission and involuntary admission).

(*ii*) *Potential moderators of outcome:* We investigated interaction effects on the intervention outcome for voluntary patients and involuntarily admitted patients and two other variables identified *a priori* to be associated with VOICE (Wing & Brown, 1970; Evans *et al.* 2012; Csipke *et al.* 2016) (gender, ethnicity).

(*iii*) *Activities and perceived contacts:* We first compared the average numbers of activities and numbers of participants before and after the intervention and accounted for ward effects using a fixed effects framework. Then, to corroborate the staff data, we analysed activities data collected in CITRINE using similar analyses. The fixed effects model was used to take account of ward effects. Standard errors were generated using bootstrapping with replacement due to the non-normal data distribution and we tested for the effects of potential moderators, e.g. PANSS scores.

#### Intervention costs and changes in resource allocation costs

The training costs associated were estimated as the cost of employing a clinical psychologist to lead the training and the opportunity cost of nurses and occupational therapists attending training. In order to calculate cost per patient week, we assumed that the longevity of the treatment was equal to the average follow-up time in the trial. While the intervention did not alter the resources allocated to the wards, we investigated how the composition and frequency of perceived staff contacts changed by multiplying the service use information collected using CITRINE by the respective salaries and used the total cost as a summary measure. Our regression analysis followed the same format as the analysis of patient data, but to allow for skewness and kurtosis we calculated bootstrapped standard errors.

All analyses were carried out using Stata versions 11 and 12.

### Patient involvement

The study was designed with the help of service users who were also involved in the study design, implementation, analysis and dissemination of the results, e.g. in the design of the primary outcome (Evans *et al.* 2012).

## Results

Data were available from 1108 participants who took part either before or after the intervention and were 70% of the population eligible to participate at the time of the assessments (see CONSORT diagram in Table 1). A total of 1058 (95.5%) individuals provided enough data for the analysis of the primary outcome. The characteristics of the patients in the wards were not different in the pre- and post- intervention samples (see Table 2 and online Web Table 2 for each of the 16 wards). The intervention consisted of 826 staff attending training sessions with a mean per ward of 51.6 staff attending (s.d. 19.4). The number of sessions varied depending on staff available on the ward (range 24–81 sessions).

**Table 1.** Study population and recruitment (CONSORT)

		Number of patients	
Time period		Pre-intervention wards	Post-intervention wards
1	Total on wards	446	–
	Not eligible	203	–
	Refused consent	70	–
	**Total consented**	**172**	–
2	Total on wards	380	84
	Not eligible	177	41
	Refused consent	48	9
	**Total consented**	**155**	**34**
3	Total on wards	274	132
	Not eligible	134	65
	Refused consent	39	13
	**Total consented**	**101**	**54**
4	Total on wards	233	204
	Not eligible	128	85
	Refused consent	42	35
	**Total consented**	**63**	**84**
5	Total on wards	127	234
	Not eligible	43	73
	Refused consent	31	71
	**Total consented**	**53**	**90**
6	Total on wards	102	59
	Not eligible	43	19
	Refused consent	23	14
	**Total consented**	**36**	**26**
7	Total on wards	155	142
	Not eligible	62	60
	Refused consent	24	20
	**Total consented**	**69**	**62**
8	Total on wards	70	88
	Not eligible	34	45
	Refused consent	15	19
	**Total consented**	**21**	**24**
9	Total on wards	–	87
	Not eligible	–	10
	Refused consent	–	13
	**Total consented**	–	**64**

**Table 2.** Demographic and clinical characteristics of participants

Description		*N* (%) or mean (s.d.)		
		Overall	Pre-intervention wards	Post-intervention wards
		*1108*	*670*	*438*
**Demographic characteristics**				
Gender	Men	609 (55%)	352 (53%)	257 (59%)
	Women	499 (45%)	318 (47%)	181 (41%)
Age (years)	Mean (s.d.)	39.7 (12.8)	39.7 (13.0)	39.6 (12·6)
First language	English	879 (80%)	525 (79%)	354 (82%)
	Not English	219 (20%)	141 (21%)	78 (18%)
Ethnicity	White	556 (50%)	325 (49%)	231 (53%)
	Mixed	71 (6.4%)	38 (5.7%)	33 (7.5%)
	Asian	59 (5.3%)	30 (4.5%)	29 (6.6%)
	Black	377 (34%)	250 (37%)	127 (29%)
	Chinese	3 (0·3%)	3 (0·5%)	–
	Other	41 (3.7%)	23 (3.4%)	18 (4.1%)
Legal status of admission	Involuntary	616 (56%)	386 (58%)	230 (53%)
	Voluntary	485 (44%)	280 (42%)	205 (47%)
Number of previous admissions	Mean (s.d.)	3.6 (5.5)	3.6 (6.0)	3.5 (4.7)
Primary clinical diagnosis	Psychosis	513 (48%)	330 (50%)	183 (45%)
	Other	554 (52%)	329 (50%)	225 (55%)
Length of stay (days)	Mean (s.d.)	34.4 (53.8)	37.7 (61.7)	29.4 (38.5)
**Measured clinical outcomes**				
VOICE potential range 19–114		54.5 (18.0)	56.5 (19.1)	54.2 (17.2)
SS-RES		89.9 (26.8)	91.3 (27.1)	86.4 (24.2)
PANSS		53.5 (15.3)	55.6 (15.8)	52.1 (13.1)
NOSIE		15.8 (8.2)	16.2 (8.5)	15.4 (7.3)

### Primary outcome – Did patients’ perceptions of care improve following the staff training intervention?

A total of 644 service users provided data pre-intervention and 414 post-intervention. A regression model adjusting only for ward and time estimated the standardised intervention benefit as 0.19 (mean VOICE score pre-intervention = 56.5, s.d. = 19.1, *n* = 644; mean post-intervention = 54.2, s.d. = 17.2, *n* = 414). The only confounder identified was legal status (an *a prior* moderator) and the adjusted model provides weak evidence for benefit (standardised effect −0.18, 95% CI 0.38 improvement to 0.01 deterioration, *p* = 0.062). We found two other effects (independent of treatment or ward); a deterioration in VOICE score over time by 0.06 s.d. per month (95% CI 0.01–0.12; *p* = 0.021) and, over the whole trial, voluntary patients were more positive about the ward environment than involuntary patients by 0.27 s.d. (95% CI −0.40 to −0.15, *p* < 0.0001).

#### Effect of coercion admission status (voluntary v. involuntary) and other potential moderators

There was a significant interaction only with legal status (*p* = 0.006), with good evidence that the intervention improves VOICE scores of people admitted involuntarily [standardised improvement of −0.35 (95% CI −0.12 to −0.57, *p* = 0.002)]. Among people in hospital voluntarily we found no evidence of an intervention effect (standardised effect = −0.01, 95% CI −0.23 to 0.22, *p* = 0.955).

### Secondary outcome: satisfaction (SSS-RES)

A total of 1032 patients completed the measure [625 on pre-intervention wards (mean 91.3, s.d. = 27.1) and 407 on post-intervention wards (mean 86.4, s.d. = 24.2)]. A linear regression model suggested an intervention benefit of 4.15 points (95% CI −9.22 to 0.92; *p* = 0.109). As with the VOICE measure, there is good evidence (*p* = 0.005) for an interaction effect with legal status. For those who are compelled to accept treatment, the intervention benefit was estimated as −8.44 (95% CI −14.36 to −2.52; *p* = 0.005) but no evidence of a treatment effect in voluntary patients (0.61; 95% CI −5.39 to 6.60; *p* = 0.842).

### Changes in resources

#### Ward activities

Using ward records, the mean number of activities increased post-intervention by 1.5 (95% CI −0.4 to 3.4, *p* = 0.121) from 6.3 to 7.8 and the average number of people attending increased by 6.3 (95% CI −4.1 to 16.6, *p* = 0.226) from 29.7. Of those patients who consented to be in our study there were increases in the average number of different activities attended following the staff training (from 2.14 activities by 0.59, 95% CI 0.02–1.22, *p* = 0.059) and in the number of sessions attended (from 4.14 session by 0.68, 95% CI −0.67 to 2.13, *p* = 0.320). There was no effect of any potential moderators including patient symptoms.

### Costs

#### Intervention costs

The total training cost was approximately £ 1 56 000, amounting to £10 per patient per week given an average number of 18 patients per ward and an average post-intervention follow-up of 55 weeks (online web Table 2). Eighty per cent of this cost was due to the opportunity cost of nurses attending the training sessions.

#### Changes in resource allocation

The intervention resulted in increases in the cost summary for patient viewed meaningful contacts amounting to £12 per patient (95% CI −£76 to £98, *p* value: 0.774). There were no significant interactions of the intervention with potential moderators (PANSS scores, no of previous admissions, legal status and ethnicity).

### Secondary effects on symptoms and behaviour

The means for both patient informed symptom ratings (PANSS) and Nurse rated behaviour (NOSIE) suggest improvements over time (see Table 2, online web Table 1), but neither was significant even after adjusting for potential confounders.

## Discussion

We endeavoured to achieve improved perceptions of inpatient care in a sustainable way using a simple staff training programme for various evidence-based therapeutic activities. We believe that this training could have a number of beneficial effects on staff morale and confidence in their therapeutic skills that could well go beyond the delivery of any particular activity. Although there was only tentative evidence of an overall effect, we discovered a significant benefit for an important target group – those admitted under legal sanction. The wards we studied were representative of many serving urban and inner city areas with varied background socioeconomic factors but similar patient diagnostic characteristics and chronicity to those found in most mental health wards. We therefore have no reason to assume that the effects of staff training would be much different in other areas.

Our results were further validated by the significant positive effect on satisfaction, again for those patients who were legally detained. We achieved these effects despite staff training having only a modest impact on the day to day life of the wards. The extra costs of implementing the intervention were modest, amounting to £10 per patient per week. Although other costs were not increased at the ward level, there was a realignment following the intervention with patients receiving care that cost £12/week more. Whether these extra costs are justified or not depends on the value placed on improving patient perceptions among those legally detained.

The impact on wards is not unexpected since the association between social interaction, taking part in therapeutic activities and their impact on patient behaviour mirrors the effects of changes to mental health institutions in the 1960s (Wing & Brown, 1970). Activities break up the monotony on wards (Walsh & Boyle, 2009) and provide a forum for patient interaction (Csipke *et al.* 2016). Crucially they also distinguish a therapeutic environment from one that is purely about incarceration. All our wards already had a weekly activity schedule largely comprising activities such as cookery or bingo, that, while valued (Star Wards, 2014), were not evidence based therapeutic interventions advocated as best practice (e.g. Sainsbury Centre for Mental Health, 2004; Walsh & Boyle, 2009; NICE, 2010) or likely to be perceived as such by patients. Our nurses were trained to deliver a number of evidence-based activities, but we had no control over the number of sessions that were run. Patient reported activities did increase with some groups replacing existing non-evidence-based ones and the number of valued contacts and activities did increase at little cost. In light of that, increasing the mean number of activities from 6 to 8 may be considered a success.

The most important impact is on patients themselves. Previous studies demonstrated deterioration in inpatient care as viewed by patients (Wing & Brown, 1970) and this was noticeable in this study. It is unclear why this has been the case, but there are links to shorter hospital stays, compulsory admissions and increased disturbance (Laker *et al.* 2012; Csipke *et al.* 2014; Williams *et al.* 2014). It is therefore gratifying that there was a measurable, albeit subjective, benefit for those patients who were legally detained thus bucking the general trend. Patients who agree to accept inpatient treatment already consider the ward to be of therapeutic value so they are unlikely to view the modest impact on the wards as a large improvement. Those who are legally detained are more critical of inpatient services. Changing their views is very important as it may affect future interactions with mental health services and perhaps even the potential to avert a future compulsory admission (Laker *et al.* 2012; Theodoridou *et al.* 2012; Care Quality Commission, 2014, 2015; van der Post *et al.* 2014). Viewing the ward as more therapeutic may also affect recovery, but this study was not designed to test this possibility.

An often repeated justification by ward staff for not running activities is that people are too ill to take part. Symptom severity did not influence increased group attendance suggesting that patients can benefit from the provision of increased activities even if they have higher symptoms.

Ward staff were given choices about what they provided so that the intervention training fitted their patient mix, their current activities and their skills set. Both these factors ensured that the interventions were more valued, clinically useful and feasible for long-term use.

## Strengths and limitations

We tested the impact of training in highly charged mental health services in two NHS trusts situated in both poor and more affluent areas. The strengths of the study included measuring the intervention effects through large-scale patient evaluation and involved more than 70% of the eligible population. In addition, participant characteristics were tested as potential individual patient confounders and controlled in the analyses. Finally, the training provided was available in the NHS. However, we do not know if every patient would benefit from the intervention as not everyone on the ward who was exposed to the intervention consented or was eligible to take part. We do know that the benefits were not affected by the severity of symptoms. So we do not know what proportion of patients on those wards will benefit or the way to increase the likelihood of those benefits.

A further strength was to examine the longer term impact of these interventions when implementation was under staff control as suggested in the MRC process evaluation model for complex interventions (Theodoridou *et al.* 2012). A limitation is that we analysed only the intention-to-treat effects of providing an intervention (training staff) on the outcome (change in patient perceptions of the ward). But there are likely to be more routes to improved patient perceptions, including the effect of the activities themselves on a patient's sense of wellbeing. We did not assess any single intervention, so we do not know if some were more effective than others and we did not test the effects of individual exposure. Rather we measured the effects of a simple package of training which provided activity opportunities, but patients were not obliged to attend.

One of the most common complaints about inpatient services is the extreme boredom and lack of therapeutic activities occurring on the wards (e.g. Wing & Brown, 1970; Walsh & Boyle, 2009; Theodoridou *et al.* 2012; Care Quality Commission, 2014, 2015; Csipke *et al.* 2016). Participation in activities was related to more positive perceptions of the wards demonstrating that they can be a much valued component of inpatient services regardless of illness severity. This belies the belief that acutely ill people cannot take part in meaningful activities and supports the view that more therapeutic activities could be of value and are appreciated.

In conclusion, we discovered that with only a relatively small amount of investment in training for inpatient staff it was possible to measure improvements in the views of those who were coerced into receiving inpatient care through involuntary admissions even some considerable time after the intervention had been introduced. The effect of improved quality of care has now been linked to patient views for the first time. There is no evidence as yet that we had an effect on those voluntarily admitted, but it is possible that there are other mediators or moderators of the relationship between the intervention and the outcome that might explain this current lack of direct effects. The improvement for those involuntarily admitted was produced with little effect on the costs of care. We speculate that other potential gains following the improved patient perceptions may be better engagement with mental health services in the involuntarily admitted group. Using data gathered in an earlier study, if the better engagement results in only seven patients (95% CI 6–8, <1% of the current sample) agreeing to a voluntary rather than an involuntary admission then the cost of the training programme would be covered (Moore *et al.* 2015) by the savings from shorter admissions. This will be investigated in future analyses. This is the first reported significant method for improving the inpatient experience since the UK Francis Report (Francis, 2013) and shows that with some investment it is possible to improve mental health patients’ views of their care – particularly those who clearly have not had a rosy view and therefore were coerced into receiving that care through legal detention in hospital.

## Supplementary material

For supplementary material accompanying this paper visit http://dx.doi.org/10.1017/S003329171700188X. click here to view supplementary material
