# Supplementary material for: Improving patient experiences of mental health inpatient care: a randomised controlled trial
Source: Psychol Med. 2017 Jul 20;48(3):488–97. doi: 10.1017/S003329171700188X (PMC5757411; doi:10.1017/S003329171700188X)
Supplement: Supplementary file 1 [file S003329171700188Xsup001.docx]

**Web Table 1**

**The Intervention - Staff Training**

For more detail on the training guidance including training materials see http://www.perceive.iop.kcl.ac.uk/doorways.html

|  | **Format** | **Staff training requirement** | **Time required for implementation** |
| --- | --- | --- | --- |
| Communication skills: “Working Together” | Training session for staff, covering two main components:  (i) therapeutic communication within the CBT model.  (ii) understanding and avoiding aggression.  Training delivered with a service user trainer. | 1 x 3-hour training sessions.  More than 50% of ward staff should attend. | N/A. This is a general approach to working with service users, staff are not expected to implement any specific group. |
| Social Cognition & Interaction Training | 3 session group. Intervention intended to improve peoples' social skills. Aims are to help people avoid misunderstandings with others by understanding social situations better. | 2 x 1-hour training sessions | 3 x 45 min sessions. Group to be run every day for 1 week followed by 2 weeks off. |
| Medication Education | One off group, plus 1-to-1 sessions. Pharmacist to run groups designed to increase knowledge and insight about medication, in addition to offering 1-to-1s to discuss individual concerns. | 1x2 hour training session with ward pharmacist | A single 45 min group delivered once per week. Individual 1 hour sessions offered as needed. |
| Cognitive Remediation Therapy | CIRCuiTS computer-based package. 1-1 approach designed to address the cognitive deficits seen in people with schizophrenia, such as problems with memory, concentration and organisation. | 1 x 3-hour training introduction and manual with explanation of the remit. Provided to ward OT(s) | N/A. To be carried out by ward OT as permitted by other duties. |
| Hearing Voices Group | 3 session group. Intervention based on CBT, intended to reduce distress associated with hearing voices, learn new coping skills and improve self-esteem. | 1 x 2-hour training session | 3 x 45 min groups. Group to be run every day for 1 week followed by 2 weeks off. |
| Self-esteem & Coping with Stigma | 3 session group. Intervention based on CBT, focusing on the stigma associated with mental health problems, including the negative self-evaluations which may maintain low self-esteem. | 1 x 2-hour training session. | 3 x 45 min groups. Group to be run every day for 1 week followed by 2 weeks off. |
| Emotional Coping Skills | 3 session group. Intervention based on Dialectical Behaviour Therapy, incorporating elements of mindfulness. Teaches skills for coping with overwhelming negative emotions, particularly suitable for people who self-harm. | 1 x 2-hour training session. | 3 x 45 min groups. Group to be run every day for 1 week followed by 2 weeks off. |
| Problem Solving Skills | 1 off group. Teaches a structured method for problems solving, involving identifying the problem, brain storming possible solutions, and selecting the best solution(s) | 1 x 2-hour training session. | 1 x 45 min group delivered once per week. |
| Relaxation techniques / “Chill-out” group | 1 off group. Teaching progressive muscle relaxation, breathing exercises. If run in the evening this can be good preparation for sleep, i.e. winding down before bed, reducing caffeine intake. | 1 x 2-hour training session. | 1 x 45 min group delivered once per week. |

| **Web table 2: Baseline characteristics of participants, per ward** | | | | | | | | | | | | | | | | | | | | | | |
| --- | --- | --- | --- | --- | --- | --- | --- | --- | --- | --- | --- | --- | --- | --- | --- | --- | --- | --- | --- | --- | --- | --- |
|  | Mean (standard deviation) or number (percentage) of baseline characteristic | | | | | | | | | | | | | | | | | | | | | |
| Ward | 1  N=47 | 2  N=104 | 3  N=36 | 4  N=66 | 5  N=73 | 6  N=71 | 7  N=57 | 8  N=93 | 9  N=83 | 10  N=81 | 11  N=106 | 12  N=113 | | 13  N=52 | | | 14  N=48 | | 15  N=41 | | 16  N=37 | |
| **Demographic and clinical history information** | | | | | | | | | | | | | | | | | | | | | | |
| Female | 0 (0) | 0 (0) | 36 (100) | 66 (100) | 0 (0) | 71 (100) | 57 (100) | 0 (0) | 83 (100) | 81 (0) | 48 (45∙3) | 52 (46∙0) | | | 25 (48∙1) | | 26 (54∙2) | 18 (43∙9) | | 16 (43∙2) | | |
| Age (years) | 42∙4 (11·6) | 36∙7 (11·3) | 38∙6 (10·5) | 42∙2 (10·8) | 41∙6 (11·9) | 39∙2 (14·8) | 42∙8 (12·5) | 39∙2 (10·6) | 46∙4 (13·5) | 42∙6 (12·7) | 28∙1 (9·0) | 42∙9 (12·8) | | | 42∙8 (14·5) | | 39∙6 (12·5) | 34∙7 (12·8) | | 38∙1 (9·6) | | |
| First language English | 35 (76∙1) | 92 (89∙3) | 33 (91∙7) | 49 (74∙2) | 55 (75∙3) | 56 (78∙9) | 43 (79∙6) | 66 (71∙0) | 69 (84∙2) | 62 (76∙5) | 81 (76∙4) | 78 (70∙3) | | | 52 (100) | | 42 (87∙5) | 29 (74∙4) | | 37 (100) | | |
| Ethnicity: |  |  |  |  |  |  |  |  |  |  |  |  | | |  | |  |  | |  | | |
| white | 25 (53·2) | 47 (45·2) | 26 (72·2) | 39 (59·1) | 37 (50·7) | 36 (50·7) | 35 (61·4) | 31 (33·3) | 32 (38·6) | 39 (48·2) | 30 (28·3) | 52 (46·4) | | | 44 (84·6) | | 27 (56·3) | 22 (53·7) | | 34 (91·9) | | |
| black | 19 (40·4) | 34 (32·7) | 3 (8·3) | 19 (28·8) | 23 (31·5) | 23 (32·4) | 18 (31·6) | 50 (53·8) | 33 (39·8) | 32 (39·5) | 54 (50·9) | 41 (36·6) | | | 3 (5·8) | | 14 (29·2) | 8 (19·5) | | 3 (8·1) | | |
| Detained under Mental Health Act | 28 (59∙6) | 59 (57∙3) | 3 (8∙3) | 39 (59∙1) | 50 (68∙5) | 46 (64∙8) | 38 (70∙4) | 62 (66∙7) | 57 (68∙7) | 66 (81∙5) | 65 (61∙9) | 53 (47∙3) | | | 13 (25∙0) | | 19 (39∙6) | 14 (35∙0) | | 4 (10∙8) | | |
| Number of previous admissions | 5∙3 (6∙0) | 2∙2 (3∙3) | 2∙3 (2∙8) | 3∙4 (5∙1) | 3∙7 (3∙4) | 4∙1 (6∙1) | 4∙2 (5∙4) | 3∙3 (3∙6) | 4∙5 (5∙4) | 3∙7 (4∙4) | 1∙7 (2∙4) | 4∙1 (10∙3) | | | 3∙4 (3∙5) | | 6∙4 (8∙3) | 3∙9 (4∙7) | | 3∙3 (3∙5) | | |
| Primary diagnosis of Psychosis | 28 (59∙6) | 44 (43∙6) | 3 (8∙3) | 32 (49∙2) | 39 (55∙7) | 26 (36∙6) | 21 (41∙2) | 54 (58∙7) | 40 (49∙4) | 42 (53∙9) | 65 (63∙7) | 51 (50∙5) | | | 14 (26∙9) | | 22 (46∙8) | 17 (44∙7) | | 15 (42∙9) | | |
| Length of stay (days) | 56∙4 (90∙7) | 36∙2 (71∙0) | 31∙9 (36∙9) | 26∙1 (26∙3) | 32∙5 (52∙9) | 29∙7 (33∙0) | 30∙9 (32∙5) | 48∙4 (68∙8) | 37∙0 (36∙0) | 44∙7 (83∙1) | 27∙6 (26∙1) | 21∙9 (37∙2) | | | 26∙4 (31∙7) | | 43∙4 (84∙3) | 31∙3 (35∙6) | | 34∙3 (30∙4) | | |
| **Clinical characteristics*** | | | | | | | | | | | | | | | | | | | | | | |
| VOICE | 53∙6 (18∙2) | 52∙4 (19∙2) | 35∙5 (10∙2) | 55∙3 (15∙7) | 57∙7 (21∙1) | 53∙3 (14∙5) | 57∙2 (14∙4) | 61∙4 (20∙3) | 63∙5 (19∙5) | 54∙2 (19∙9) | 51∙3 (18∙1) | 55∙7 (20∙6) | 48∙3 (17∙7) | | | 52∙9 (11∙9) | | 51∙3 (12∙5) | | 48∙6 (16∙9) | |  |
| Age | 43∙5 (14∙0) | 35∙4 (12∙4) | 35∙1 (12∙7) | 42∙1 (13∙6) | 40∙3 (14∙8) | 38∙2 (13∙8) | 45∙9 (10∙8) | 39∙7 (6∙7) | 40∙0 (12∙0) | 39∙9 (11∙5) | 31∙2 (10∙4) | 50∙3 (12∙6) | 49∙2 (12∙2) | | | 39∙1 (12∙2) | | 29∙6 (6∙4) | | 34∙0 (10∙4) | |  |
| Gender (% female) | 0 | 0 | 100 | 100 | 0 | 100 | 100 | 0 | 100 | 0 | 44∙8 | 41∙7 | 55∙6 | | | 66∙7 | | 38∙9 | | 33∙3 | |  |
| SSS-RES | 83∙3 (27∙6) | 92∙8 (23∙5) | 71∙1 (12∙6) | 93∙9 (26∙6) | 86∙2 (28∙4) | 88∙3 (21∙8) | 102∙0 (27∙3) | 98∙6 (28∙6) | 100∙7 (24∙0) | 89∙4 (29∙0) | 86∙1 (30∙0) | 96∙1 (30∙3) | 78∙0 (29∙9) | | | 87∙4 (18∙9) | | 82∙7 (19∙0) | | 83∙0 (27∙9 | |  |
| PANSS | 49∙6 (8.2) | 58∙5 (20.6) | 55∙4 (10.8) | 58∙3 (15.8) | 54∙5 (8.9) | 54∙0 (12.1) | 56∙0 (16.3) | 54∙1 (11.1) | 69∙6 (18.8) | 40∙7 (4.2) | 66∙7 (20.3) | 40∙8 (6.0) | 62∙1 (12.8) | | | 45∙6 (12.4) | | 46∙3 (11.2) | | 42∙3 (8.4) | |  |
| NOSIE | 19∙5 (7∙7) | 16∙6 (9∙4) | 14∙6 (9∙5) | 16∙8 (5∙9) | 21∙2 (8∙6) | 14∙4 (7∙1) | 15∙6 (9∙2) | 15∙7 (10∙5) | 16∙0 (7∙8) | 20∙8 (8∙1) | 12∙2 (6∙5) | 15∙7 (7∙7) | 11∙5 (7∙7) | | | 14∙0 (8∙3) | | 16∙3 (6∙8) | | 14∙3 (4∙7) | |  |
| Change in HONOS | -2∙0 (6·7) | -2∙1 (5·4) | -4∙4 (5·8) | -7∙2 (10·7) | -5∙4 (4·9) | -3∙8 (7·8) | -3∙3 (11·0) | -3∙5 (5·3) | -7∙0 (7·7) | -3∙2 (10·3) | -5∙1 (6·0) | -3∙1 (6·2) | - | | | - | | - | | - | |  |
| GAF | 49∙6 (8·3) | 40∙9 (9·7) | 48∙8 (4·9) | 39∙9 (9·8) | 39∙0 (7·9) | 39∙6 (9·6) | 43∙6 (9·6) | 36∙2 (10·4) | 40∙3 (12·1) | 39∙3 (13·1) | 43∙3 (13·0) | 37∙7 (17·0) | 42∙1 (10·8) | | | 47∙0 (7·8) | | 54∙4 (12·5) | | 51∙7 (14·6) | |  |
